# Hydroclimate variability in the central Mediterranean during MIS 17 interglacial (Middle Pleistocene) highlights timing offset with monsoon activity

**DOI:** 10.1038/s41598-023-45812-x

**Published:** 2023-11-02

**Authors:** Luca Capraro, Alessandro Incarbona, Eliana Fornaciari, Nadia Sabatino, Stéphane Scaillet, Rodolfo Sprovieri, Mario Sprovieri

**Affiliations:** 1 Dipartimento di Geoscienze, Università degli Studi di Padova, Via G. Gradenigo 6, 35131 Padova, Italy; 2 Dipartimento di Scienze della Terra e del Mare, Università degli Studi di Palermo, Via Archirafi 22, 90123 Palermo, Italy; 3 National Biodiversity Future Center (NBFC), Piazza Marina 61, 90133 Palermo, Italy; 4 Istituto per lo studio degli impatti Antropici e Sostenibilità in ambiente marino, Consiglio Nazionale delle Ricerche, Via del Mare 3, 91021 Torretta Granitola, Campobello di Mazara, Trapani Italy; 5 CNRS, Institut des Sciences de la Terre d’Orléans (ISTO), 1A rue de la Férollerie, 45071 Orléans, France

**Keywords:** Climate change, Palaeoceanography, Palaeoclimate, Climate sciences, Ocean sciences

## Abstract

Mediterranean climates are characterized by warm, dry summers and mild, rainy winters. Previous studies suggest that over the last 1.36 Myr, Mediterranean winter rainfalls were in phase with the African monsoon. Here we present a high-resolution terrestrial and marine dataset for the Marine Isotope Stage 17 interglacial (Middle Pleistocene) from Southern Italy, showing that precipitation rates and regimes in the central Mediterranean varied independently of the monsoon system. Specifically, events of extreme summer precipitation were promoted by increased regional insolation rates and/or extratropical cyclones, and their magnitude was further enhanced by the advection of cool and humid North Atlantic air during stadials. Our findings provide new information on the short- to mid-term natural hydroclimatic variability of the Mediterranean basin, and offer new critical insights on land–ocean interactions at the regional scale by complementing previous analyses on the displacement of storm tracks toward southern Europe.

## Introduction

Mediterranean climate is characterized by mild wet winters and warm to hot, dry summers. In the Mediterranean basin, summer droughts are secured by the northward migration of North African and Azorean highs, hampering the influx of Atlantic storm tracks. As anticyclones retreat in the fall, westerlies and polar-continental air masses yield abundant winter precipitation^1–3^. Regional rainfall rates also respond to the North Atlantic Oscillation (NAO)^4,5^. Previous studies emphasized the importance of winter precipitation in shaping the long-term environmental evolution of the northern Mediterranean and Balkan borderlands in the recent geological past. It is assumed that the magnitude of interglacial winter precipitation over the last 1.36 Myr varied in phase with the African monsoon, as the low-latitude insolation forcing on Mediterranean sea-surface temperatures would strengthen the local cyclogenesis and fuel winter storm tracks from North Atlantic low-pressure systems^6–8^. However, small-scale temporal relationships between African Monsoon and Mediterranean precipitation are still poorly understood. Holocene studies question the synchronicity between the two, because the advection of humid Atlantic air over the southern Mediterranean increased regional precipitation since the middle Holocene, considerably after the beginning of the Green Sahara event^9–13^.

Our study record, which we refer to as the Blatta section (39° 0′ 37.07″ N, 16° 50′ 31.57″ E), is a ca. 23 m-long mid- to outer-shelf Lower to Middle Pleistocene marine succession exposed in the northwestern sector of the Crotone Basin (Southern Italy). It is part of an expanded, shallowing-upward stack of slope to inner shelf sediments encompassing continuously the MIS 26-MIS 16 interval (Supplementary Material 1)^14,15^. The Blatta section straddles MIS 17, a poorly investigated interglacial if compared to the contiguous MIS 19 and MIS 15-MIS 13, or other recent integlacials^16,17^. MIS 17 investigation is especially relevant considering that the establishment of 100-kyr glacial cycles took place shortly after^18,19^. A steady sampling pace of 10 cm was followed across the MIS 17 interval (Supplementary Material 1). The very high resolution and sensitivity of land-sea geochemical and micropaleontological proxies employed for this work shed light on past seasonal hydroclimate variability in the central Mediterranean, which is a crucial information for understanding present and future environmental changes^3^.

### Chronology of the Blatta section

Our age model is based on a linear interpolation between 11 tie-points identified as ‘cold spells’ in the Blatta section, corresponding to “heavy” δ^18^O values for *U. peregrina* and/or spikes of *N. pachyderma* sinistral coiling (sx) (Supplementary Material S2, Fig. S2). Short-lived abundance fluctuations of other planktonic foraminifera species, such as the warm water species *Globigerinoides ruber*^20,21^, confirm the presence of a pervasive millennial-scale variability throughout the record (Supplementary Material S3). Target record is the coeval succession of stadial events at the Iberian Margin^22^ (8-IMS1 and 7-IMS10 to 7-IMS19), where the signal of sub-orbital oscillations in Atlantic Meridional Overturning Circulation (AMOC) strength is sharp^23–28^. It is thus assumed that AMOC slowdown led to a southward shift of the Polar Front and increases in the strength of the Northern Hemisphere atmospheric activity, analogous to the stadials and Heinrich events of the last glacial that are associated with a drop in Mediterranean SSTs, incursions of *N. pachyderma* through the Gibraltar Strait and increased ventilation of the Mediterranean seafloor^24–26,28^.

Correlation is further validated by the overall match between our benthic δ^18^O record and the δ^18^O profiles of the Iberian Margin^29^ and the LR04 benthic stack^16^ (Supplementary Material S2, Fig. S2). An age of 715.48 ± 4 ka is obtained for the Parmenide tephra, in excellent agreement with the previous ^40^Ar/^39^Ar dating of 710 ± 5 ka^15^. Calcareous nannofossils cannot provide chronology, as the identified MNN 19f. (concurrence of medium-sized *Gephyrocapsa omega* and *Pseudoemiliana lacunosa*) is a long-range Zone^30^. The calculated resolution for the main sampling pace (10 cm) is between 0.13 and 0.52 kyr, 0.38 kyr on average (Supplementary Material 2). Wide excursions in the local estimated sediment accumulation rates are consistent with the inferred mid- to outer-shelf depositional setting, where sedimentation responds to a complex interplay between sediment supply, climate, eustasy and tectonics^13,14^.

### The hydroclimate regime during MIS 17 in the Mediterranean

**Figure 1:**
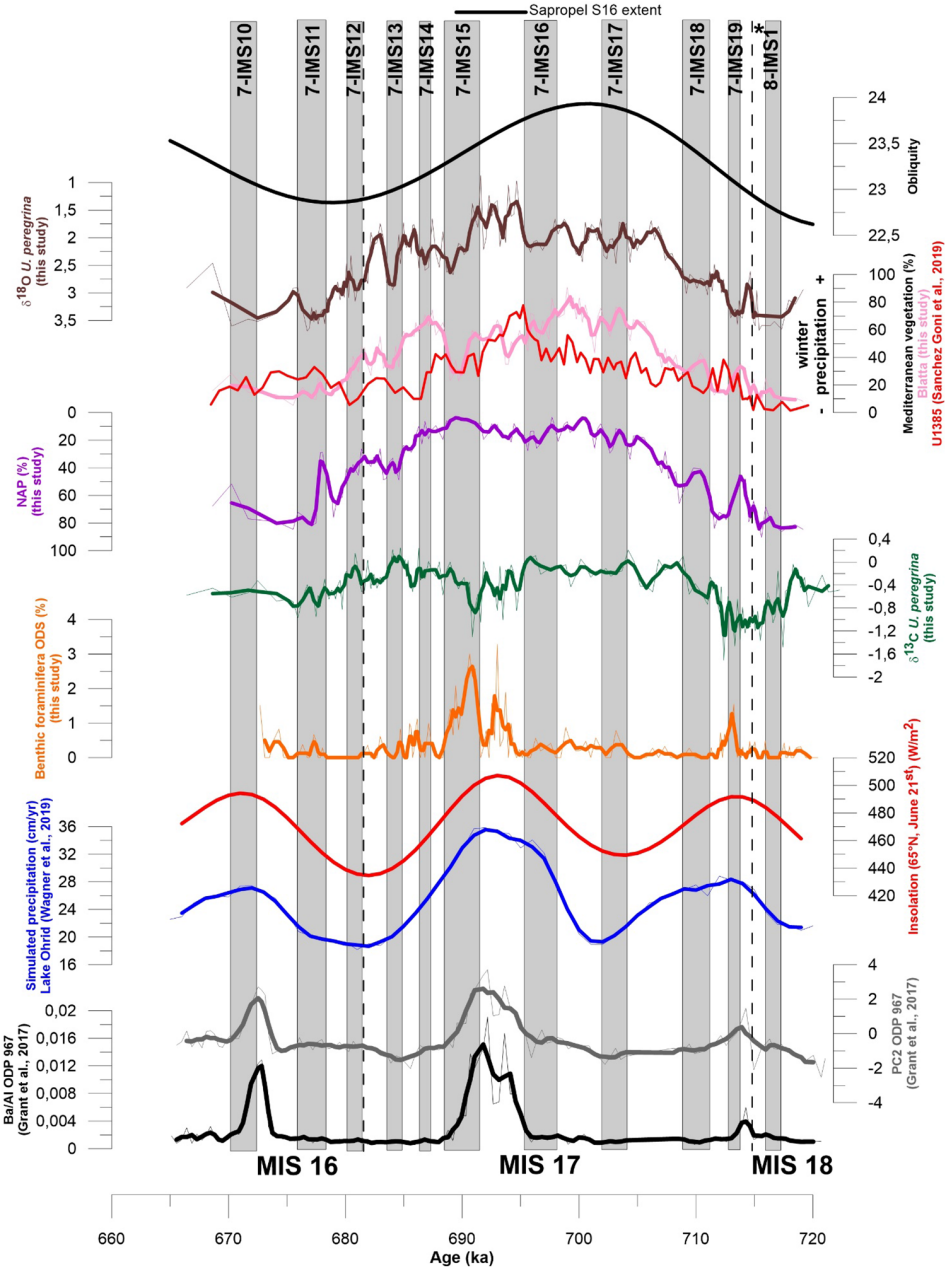
Plot of benthic δ^13^C and benthic foraminifera data at Blatta section and comparison with selected records. From the left, Ba/Al ratio and principal component 2 (PC2, river runoff) at ODP Site 967, eastern Mediterranean Sea^32^. Insolation at 65°N summer solstice variations (Laskar et al. 2004)^39^. Simulated precipitations at Lake Ohrid, Balkan region^7^. Downcore variations of *U. peregrina* δ^13^C values and ODS benthic foraminifera species percentage (this study). Non-Arboreal Pollen (NAP) (this study), note reverse axis. Mediterranean-type vegetation from Blatta section, central Mediterranean (pink line, this study) and the IODP Site U1385 (red line^18^). Downcore variations of *U. peregrina* δ^18^O values (this study). Obliquity at 65° N summer solstice variations^40^. Thick lines in ODP 967, Ohrid Lake and Blatta records are 3-pt running averages. Horizontal dotted black lines indicate MIS boundaries from ref^15^. Vertical grey boxes indicate stadial phases, progressively labeled, following the procedure by ref^19^. The timing extent of sapropel S16 follows ref^32^. The black asterisk marks the position of the Parmenide ash layer.

The magnitude of monsoonal precipitation responds to summer insolation levels in the tropics, with maximum values under conditions of minimum orbital precession and maximum eccentricity^2,31^. Although restricted to low-latitude regions, monsoonal precipitation extend their effects to the eastern Mediterranean in the form of riverine runoff, the Nile River being the largest contributor in this regard^32–34^. Extreme precession-related Nile floods are believed to cause a density-driven stratification of the eastern Mediterranean, with deposition of organic-rich layers (sapropels) in the deep domain^2,31,35^. In shallower depositional settings inadequate to sapropel formation, a geochemical signature of these events is still preserved as negative δ^18^O_planktonic_ and δ^13^C_benthic_ excursions and/or spikes in the Ba/Al ratio^2,36–38^ (Supplementary Material S4, Fig. S4). In the Blatta section, where the lithological evidence of sapropel layers is missing^15^ (see Supplementary material S5, Fig. S5), two well-defined minima in the δ^13^C record of *U. peregrina* (Fig. 1) are in chronological agreement with the peaks in Ba/Al ratio (productivity) and elemental proxy PC2 (river runoff) found in the deep eastern Mediterranean, correlative to sapropel layers S17 (around 714 ka) and S16 (around 692 ka)^35^, and with indications of increased precipitation at Lake Ohrid^7^ (Fig. 1). Concomitant increases in benthic foraminifer oxygen-deficiency stress (ODS) species (Fig. 1) confirm that the zonal vertical circulation of the northern Ionian Sea suffered from episodic slowdowns close in time to periods of precession-related maximum African monsoon activity and sapropel deposition in the eastern Mediterranean.

Terrestrial pollen from the Blatta section reveal a long-term vegetational trend that follow very closely the benthic δ^18^O record, suggesting that central Mediterranean climates responded primarily to a 41-kyr glacio-eustatic (obliquity) forcing (Fig. 1). Closed mesothermal Mediterranean-type forests were dominant during full MIS 17, pointing to present-day climatic conditions in the area, with rainy winters and dry summers^18,41,42^. Changes in the Mediterranean forest pollen record at the Blatta section follow very closely those found at the Iberian Margin IODP Site U1385, thus supporting a scenario of increased westerlies penetration into southern Europe around 695 ka^18^, with winter storm tracks reaching as far as central Mediterranean.

**Figure 2:**
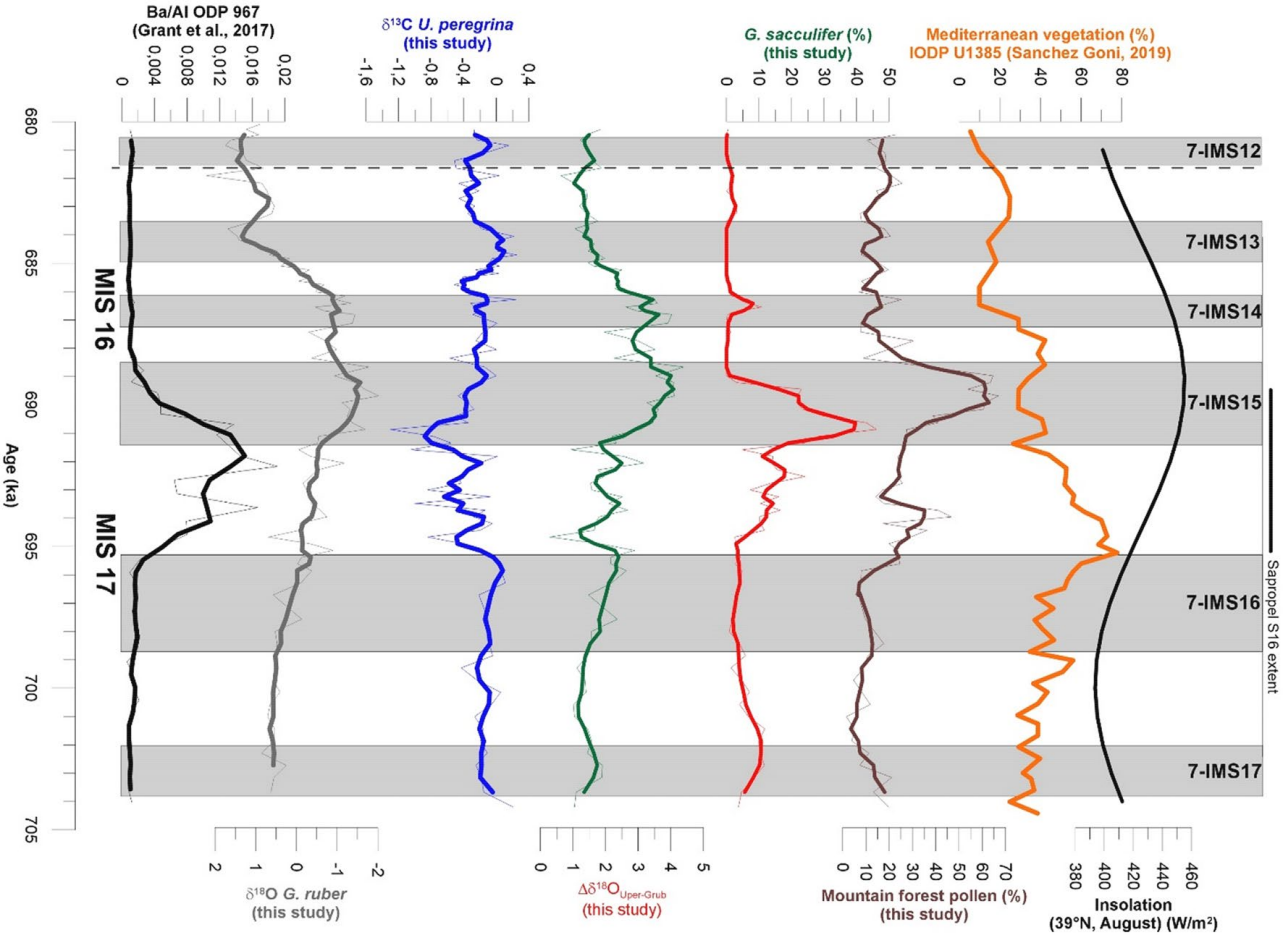
Geochemical and micropaleontological data collected at the Blatta section and comparison with selected records. From the left: Ba/Al ratio at ODP Site 967, eastern Mediterranean Sea^32^. Downcore variations of *G. ruber* δ^18^O values (this study). Downcore variations of *U. peregrina* δ^13^C values (this study). Downcore variations of Δδ^18^O values between *U. peregrina* and *G. ruber* (this study). Downcore percentage values of *G. sacculifer* (this study). Insolation at 39° N in August variations^40^. Downcore percentage values of mountain forest species (this study). Mediterranean forest from IODP Site U1385^18^. Except for the insolation record and for the Mediterranean-type vegetation, thick lines are 3-pt running averages. Horizontal dotted black lines indicate MIS boundaries from ref^15^. Vertical grey boxes indicate stadial phases, progressively labeled, following the procedure by ref^19^. The timing extent of sapropel S16 follows ref^32^.

Non-arboreal plants (NAP) communities, evocative of dry to sub-desertic climates, characterize late MIS 18 and early MIS 16 glacials (Fig. 1). Individual peaks of water-demanding conifers (Mountain Forest in Supplementary material S6, Fig. S6), suggestive of increased annual precipitation with abundant summer rainfalls, are documented within MIS 17. The main pluvial event occurs at the termination of 7-IMS15 stadial, in late full MIS 17, where water-demanding conifers attain to ca. 70% of the total pollen assemblage (Fig. 2). This episode coincides with a major decrease in *G. ruber* δ^18^O, increased *G. ruber*–*U. peregrina* δ^18^O offset (Δδ^18^O_Uper-Grub_) and abundance of *G. sacculifer* (Fig. 2), pointing to the persistence of a surface freshwater lens throughout the summer with increased density-driven stratification of the local water column^20,28^. Notably (Fig. 2), this event occurs as the Mediterranean forest declines and reconstructed summer precipitation increases in the Iberian Margin record^18^^.^

According to our age model, regional precipitation peaked ca. 3 kyr after the precession-driven monsoonal maximum and sapropel S16 deposition, as marked in our record by the negative δ^13^C excursion of *U. peregrina* (Fig. 2). A 3-kyr lag is fully consistent with the expected delay in insolation maxima between the central Mediterranean and the tropics (see August insolation at 39° N in Fig. 2). We conclude that central Mediterranean precipitation during MIS 17 were primarily controlled by obliquity but also responded to local insolation changes, which is not perfectly in phase with the precession-related African monsoon signal. Regional precipitation may have been supplied by atmospheric moisture reservoirs that changed seasonally, according to the prevailing orbital forcing at the time. Obliquity-forced winter rainfalls would exploit both Mediterranean and Atlantic sources, while precession-related precipitation would be fueled by Mediterranean yields in the late summer-early fall and by Atlantic advection in the winter^43^.

### Processes and mechanisms for increased Mediterranean summer precipitation during MIS 17

Two mechanisms may sustain a scenario of augmented summer precipitation in the central Mediterranean after sapropel S16 deposition. The first relies on the variability in local summer insolation rates, which attain a maximum during August in the study area (Fig. 2). Increased insolation rates favor the development of Convective Systems with Local Effects (CSLE) by increasing evaporation, vertical rise and condensation of humid air masses^44^. Nowadays, CSLE may cause intense precipitation in the very same area as the Crotone Basin, where steep coastal orographic barriers promote the rapid adiabatic rise of warm, humid air masses. CLSE magnitude is further increased by the periodic influx of cool air advected from the north. By comparison to recent interglacials, similar conditions may have occurred during stadials 7-IMS15 to 7-IMS13 (late full MIS 17; Fig. 2), when cooling over the Atlantic increased the frequency and magnitude of summer continental and polar outbreaks to the Mediterranean, as evidenced by drops in summer SSTs and erosion of the thermocline^17,27,45–48^.

An alternative explanation implicates the development of extra-tropical (Mediterranean) cyclones during stadial 7-IMS15. As for the previous, their magnitude is expected to increase during stadials in response to AMOC weakening. Indeed, modern summer extra-tropical cyclones are fueled by unstable air masses with high potential vorticity conveyed to the Mediterranean by the southward migration of the Polar Jet^49,50^. As documented for the Younger Dryas^40,51,52^, a similar scenario is associated with conditions of AMOC slowdown and southward expansion of sea ice, since a stronger E-W temperature gradient in the mid-latitude North Atlantic would favor the incursion of westerlies and moisture over the Mediterranean^52^.

Lake level fluctuations, vegetation biomes and speleothem isotopes in southern Europe borderlands point to an increased summer precipitation regime since the 8.2 ka event^9–11^, when the interglacial stability declined^38,53^ and continental Europe was still experiencing dryness^11,12^. CSLE described for the MIS 17 may also provide a suitable explanation for contrasting precipitation levels in Europe since the middle Holocene. By analogy with MIS 17 hydroclimate, contrasting precipitation levels may be developed between continental and southern Europe once Holocene climatic instability was established, and well later than the beginning of sapropel S1 deposition in the eastern Mediterranean (10.8 ka^2,36^). Local convective processes would explain the limited geographical (coastal) extent of the observed summer precipitation, in contrast with inner continental areas where the mechanism is ineffective^9–12,35^.

### MIS 17 Precipitation and increased stadial moisture

CSLE and extra-tropical cyclones are not mutually exclusive, as they both comply with the deployment and persistence of North Atlantic storm tracks and Mediterranean cyclogenesis^3,6–8^. However, the CSLE model better suits the overall scenario found at the Blatta section. In our record, intervals of “light” δ^18^O spikes for *G. inflata* (Supplementary Material S7, Fig. S7), higher abundances of *G. inflata* and *G. truncatulinoides* in early and late MIS 17 (Supplementary Material S3, Fig. S3) point to the development of a local cool mixed-layer^20^ in response to seasonal atmospheric low-pressure conditions. These conditions are especially well represented in stadials 7-IMS18, 7-IMS 15 and 7-IMS11, where the concomitant decline of NAP and expansion of water-demanding forest elements (Fig. S6) is evocative of increased precipitation (Fig. 1).

Increased regional precipitation during stadials seem inconsistent with other circum-Mediterranean sites, where cold spells are associated with arid climates^23^. However, we stress that the regression to semi-steppe documented during stadial periods at Lago Grande di Monticchio (southern Italy) or in the Anatolian Peninsula^23,54^, as well as the context of weaker precipitation suggested by models^55,56^, took place during a full glacial, when local convective systems were hindered by minimal local insolation rates. Our results suggest that interglacial stadials may be associated with wetter climates, differently from the classical model of dry stadials and wet interstadials that are believed to characterize glacial periods.

## Conclusions

The multi-proxy terrestrial and marine records obtained from the Blatta section point to the occurrence of suborbital climatic oscillations during MIS 17 that are consistent with the AMOC variability reconstructed at the Iberian Margin. Timing, rates and regimes of regional precipitation were out of phase with the precession-related African monsoon activity, which triggered the sedimentation of sapropel S16 in the deep eastern Mediterranean. Local summer precipitation rates increased dramatically by the end of sapropel S16 deposition, when the Mediterranean thermohaline circulation started recovering (Fig. 2), in agreement with the climatic and oceanographic evolution reconstructed across sapropel S1^11,12^. Increased summer precipitation in the central Mediterranean can be explained by the development of CSLEs and extra-tropical cyclones under the joint effects of increased local evaporation rates, stronger convection^44^ and southward shifts of the Polar Jet during stadials, which would favor the advection of cool and humid Atlantic air^52^.

Our study illustrates the importance of global climatic drivers like insolation and AMOC in impacting regional-scale hydrological systems, with amplifying effects that may have impacted the Mediterranean area during MIS 17, and possibly the early-middle Holocene. Unravelling the mechanisms and processes in place, like those here referred to as CSLEs, is a basic requisite for a better understanding and effective forecasting of extreme summer precipitation events, such as those increasingly afflicting densely populated coastal areas of the Mediterranean under the ongoing climate change.

## Methods

### Stable isotopes

Oxygen and carbon isotope analyses were run on 5/7 individuals of the planktonic foraminifera *Globigerinoides ruber* (220 samples), *Globorotalia inflata* (100 samples) and of the benthic foraminifer *U. peregrina* (250 samples). Specimens were handpicked from samples collected each 20 cm. Samples were measured by automated continuous flow carbonate preparation GasBenchII device^57^ and a ThermoElectron Delta Plus XP mass spectrometer at the IAMC-CNR (Naples) isotope geochemistry laboratory. Acidification of samples was performed at 50 °C. An internal standard (Carrara Marble with δ^18^O = − 2.43 vs. VPDB and δ^13^C = 2.43 vs. VPDB) was run every 6 samples and the NBS19 international standard was measured every 30 samples. Standard deviations of carbon and oxygen isotope measures were estimated at 0.1 and 0.08‰, respectively, on the basis of ~ 70 repeated samples. All isotope data are reported in per mil (‰) relative to the VPDB standard.

### Pollen

190 samples have been subjected to palynological investigations Rock fragments with a dry weight of 10 g were treated according to standard procedures, namely: removal of the calcite content with concentrated HCl; elimination of silicates by means of concentrated HF; digestion of the organic matter (whenever necessary) with hot diluted KOH; separation of the pollen content by means of gravitative separation with ZnCl_2_ at d = 2.004; ultrasonic disintegration of the residual inorganic fraction. Residues have been stored in glycerin and mounted at the spot on disposable slides. Pollen were analyzed under a light microscope with 100 × and 430 × magnifications. For each sample, a minimum of 195 and a maximum of 991 (average 359) grains have been counted based on the specific pollen abundance.

### Planktonic foraminifera

A total of 188 samples were prepared for planktonic foraminifera analysis. Samples were washed using a 63 µm mesh sieve and were oven-dried at 40 °C. Quantitative analysis was carried out counting all the specimens occurring on the total residue from the fraction > 125 µm.

Planktonic foraminifera species were identified following taxonomic concepts by^58,59^. The *Globigerinoides ruber* white group includes *Globigerinoides elongatus* and *Globigerinoides conglobatus*. The *Globigerinoides sacculifer* group includes *Globigerinoides trilobus* and *Globigerinoides quadrilobatus.* The *Globigerina bulloides* group includes *Globigerina falconensis*.

The planktonic/benthic (P/B) ratio, as a proxy for quantitative paleo-depth estimates, follows the calibration by ref^60^:

$${\text{D}} = {\text{e}}^{{\left( {{3}.{58718 } + \, \left( {0.{\text{3534 x }}\% {\text{P}}} \right)} \right)}}$$

where D is the seafloor depth in meters below sea level and %P is the percentage value of planktonic foraminifera specimens.

### Benthic foraminifera

A total of 188 samples were prepared for benthic foraminifera analysis. Samples were washed using a 63 µm mesh sieve and were oven-dried at 40 °C. Quantitative analysis was carried out counting all the specimens occurring on the total residue from the fraction > 125 µm.

Benthic foraminifera species were identified following taxonomic concepts by ref^61^. *Bolivina dilatata* includes *Bolivina spathulata*. *Uvigerina mediterranea* includes *U. peregrina.* Specimens of the genus *Quinqueloculina*, *Triloculina*, *Cruciloculina*, *Biloculinella*, *Miliolinella*, *Pyrgo* and *Sigmoilina* were grouped as Miliolids. The deep infaunal taxa *Chilostomella* spp. and *Globobulimina affinis* were grouped as oxygen deficiency stress (ODS) species^62^.

### Nannofossils

A total of 71 samples were investigated for calcareous nannofossils. Samples were prepared from unprocessed material as smear slides and examined using a light microscope at 1250 × magnification. Quantitative analysis was carried out counting the relative abundance of selected taxa with respect to a pristine population of at least 500 specimens^30^ with sizes ≥ 3.5 µm. Taxonomic concepts follow those of refs^63^ except for species of genus *Gephyrocapsa*, which are those of ref^64^. The adopted zonal scheme is from ref^30^.

### Ar/Ar dating

Sanidine crystals were extracted by simple disaggregation of the ash layer, with further handpicking under a binocular microscope. They were cleaned by ultrasonic etching in a dilute (2–5%) hydrofluoric solution for 3–5 min, followed by ultrasonic rinse in acetone, ethanol, and deionized water. Single crystals were individually loaded in three 4 mm I.D. holes machined into a 11 mm O.D., 3 mm thick, Al-irradiation disks. Samples were co-irradiated with the irradiation monitors ACR-2 and TCR loaded in smaller adjacent pits bracketing the three sample locations. Corrections for isotopic interferences from K, Ca, and Cl were applied using production ratios listed in Scaillet et al. (2013)^65^. After baking overnight at 180 °C, single crystals were individually analyzed with a continuous 20 W Synrad ® CO_2_ laser source coupled to a noble gas mass-spectrometer operated in pulse-counting mode^65^. Released gases were purified prior to gas admission into the mass-spectrometer by exposure for 10 min on two air-cooled GP50 S.A.E.S.® getter cartridges featuring a Zr–Al St101® alloy held at 250 °C. Age were calculated using in-house software^66^ based on conventional isotope abundances and monitor and decay constants^65^. Monitoring of the instrumental mass-fractionation is achieved by daily calibration of the atmospheric ^40^Ar/^36^Ar isotope ratio on air shots interspersed with the unknowns at different peak intensities to correct for nonlinearity and counting dead-time (for every isotope) using in-house software. Age errors are plotted and tabulated at 2σ and include corrections for (1) counting dead-time for every isotope, (2) system blanks, (3) mass-discrimination, (4) post-irradiation decay of ^39^Ar, ^37^Ar, and ^36^Cl, (5) isotope interference corrections from K, Ca and Cl, (6) atmospheric contamination, (7) neutron-flux gradients, and (8) monitor’s age error.

### Supplementary Information

Supplementary Information.

## Data Availability

The dataset produced in this study is available on the Pangaea Database.
