# Supplementary material for: Hydroclimate variability in the central Mediterranean during MIS 17 interglacial (Middle Pleistocene) highlights timing offset with monsoon activity
Source: Sci Rep. 2023 Nov 2;13:18938. doi: 10.1038/s41598-023-45812-x (PMC10622447; doi:10.1038/s41598-023-45812-x)
Supplement: Supplementary file 1 — Supplementary Information. [file 41598_2023_45812_MOESM1_ESM.docx]

Supplementary Material

Hydroclimate Variability in the central Mediterranean during MIS 17 interglacial (Middle Pleistocene) highlights timing offset with Monsoon activity

Luca Capraro^1*^, Alessandro Incarbona^2-3^, Eliana Fornaciari^1^, Nadia Sabatino^4^, Stéphane Scaillet^5^, Rodolfo Sprovieri^2^, Mario Sprovieri^4^

^1^ Università degli Studi di Padova, Dipartimento di Geoscienze, Via G. Gradenigo 6, 35131 Padova, Italy

^2^ Università degli Studi di Palermo, Dipartimento di Scienze della Terra e del Mare, Via Archirafi 22, 90123 Palermo, Italy

^3^ National Biodiversity Future Center (NBFC), Piazza Marina 61, Palermo 90133, Italy.

^4^ Consiglio Nazionale delle Ricerche, Istituto per lo studio degli impatti Antropici e Sostenibilità in ambiente marino, Via del Mare 3, 91021 Torretta Granitola, Campobello di Mazara, Trapani, Italy

^5^ CNRS, Institut des Sciences de la Terre d’Orléans (ISTO), 1A rue de la Ferollerie, 45071, Orléans, France

^*^Corresponding author, e-mail: luca.capraro@unipd.it, telephone: +39 0498279182

**Supplementary material 1: Location and Material**

The studied record, which we refer to as the “Blatta” section, is a ca. 23 m-long segment exposed in a badland area immediately south of the village of Marcedusa (Fig. S1a), in the northwestern part of the on-land Crotone Basin (39° 0'37.07"N, 16°50'31.57"E) (Fig. S1b). The “Blatta” section constitutes the upper segment of a long composite section, known as the Marcedusa composite section^1^, which epitomizes the local stratigraphy in the interval straddling the Lower-Middle Pleistocene boundary^2^. It consists in an expanded, shallowing-upward stack of slope to inner shelf sediments capped by a package of coarse-grained, shallow-water to continental sediments that herald the definitive uplift of the area. Lithologies in the open-marine part of the stratigraphy range from blue, poorly fossiliferous clayey marls, which dominate the lower and middle part of the succession, to coarse yellow sands, locally rich in shells of the cold-water bivalve *Arctica islandica*, in its upper portion^1^.

In the local succession, the youngest “true” (dark and laminated) sapropel layer occurs within the MIS 25 muds ^1^. Above, a number of dark layers containing plenty of *Chondrites* – indicative of recurring episodes of severe, yet not complete, oxygen depletion at the seafloor (“failed” sapropels) – are found in the muds of MIS 21 and 19^1^. Based on their stratigraphic distribution, these dark layers may represent the shallow-water equivalent of the sapropel layers that are found offshore the Crotone area^3^. From MIS 18 upwards, the *Chondrites*-bearing layers also disappear as the blue muds gradually transform into an amalgamated package of silty muds containing the “Blatta” section. The base of this segment is marked by a prominent ash layer (the “Parmenide ash” of ref^2^) that stands out for being stiffer and lighter in color than the embedding sediments. The interval considered for this work was investigated following a steady sampling resolution of 10 cm, with the exception of its lowermost and uppermost parts that were inspected with less detail. The outcrop was cleaned and the pristine rock exposed one meter at a time by means of a large hoe. The fresh exposure was therefore observed and described carefully, providing physical stratigraphic evidence that the depositional environment and sedimentation style remained virtually unchanged throughout the interval of relevance. A 10 cm-wide chisel was then hammered into the sediments in order to obtain evenly spaced samples, from which we extracted fragments with an average weight of ca. 300 g.


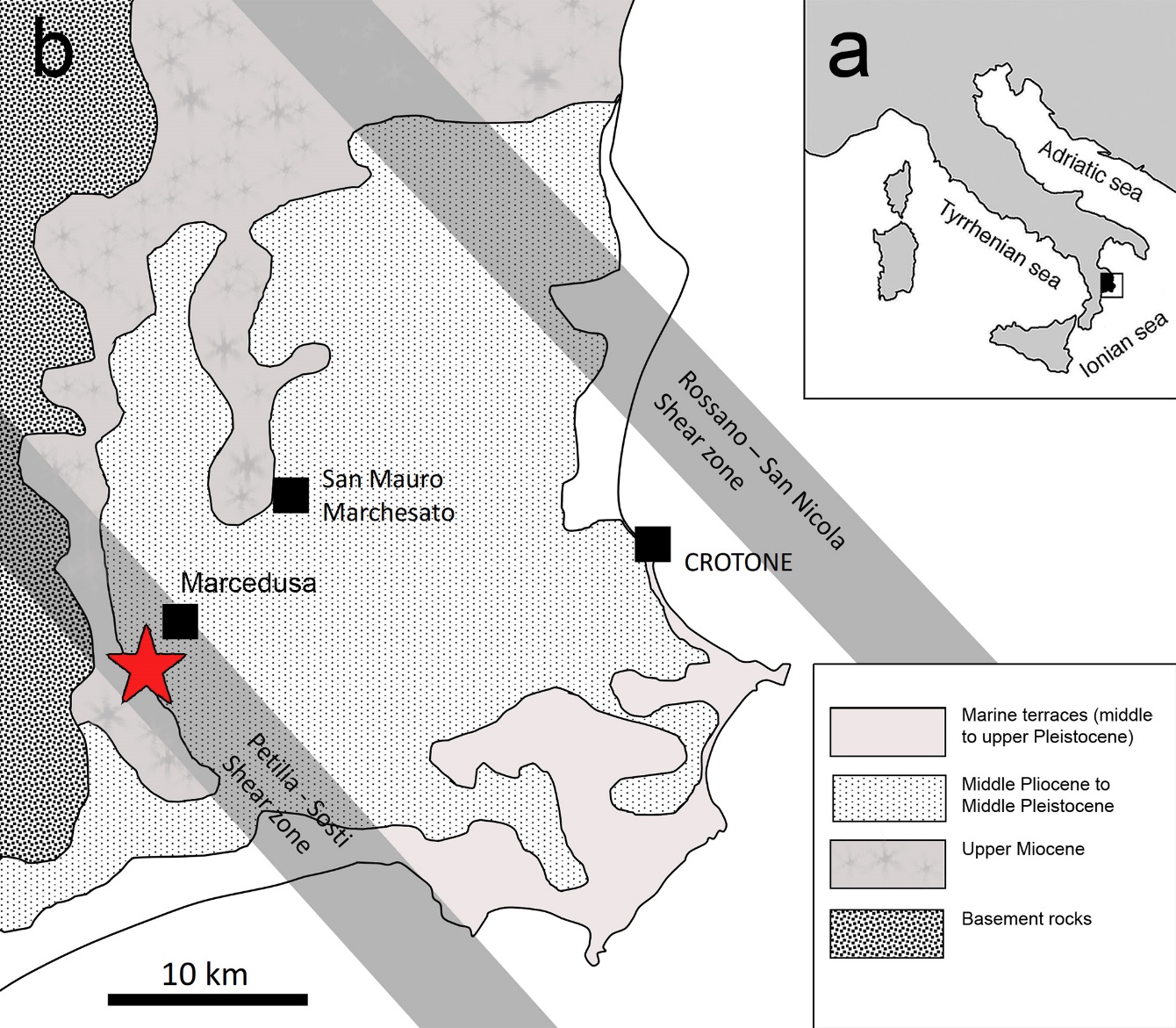


Figure S1: location map. a) position of the Crotone Basin (CB) in Southern Italy. b) simplified geological map of the Crotone Basin, with indication of the study area (red star) and main geographical references. For details, please refer to Capraro et al. (2011).

**Supplementary Material 2: Chronology**

**
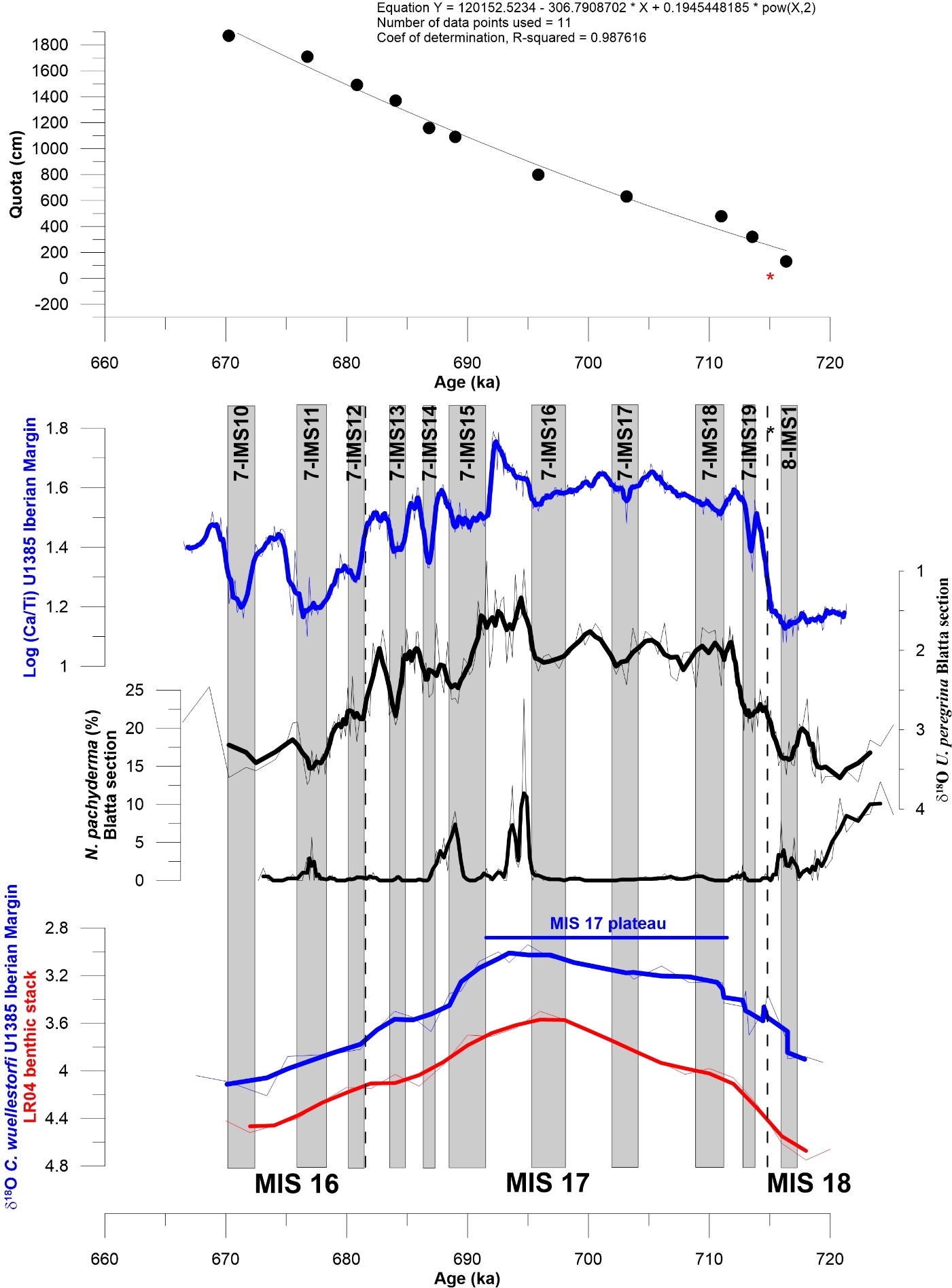
**

**Stratigraphic level (m)**

Figure S2: plot of benthic δ^18^O and planktonic foraminifera data at Blatta section and comparison with selected records for the age model assessment. From the left, LR04 benthic δ^18^O stack^4^ (red line) and benthic δ^18^O values at IODP Site U1385 Site Iberian Margin^5^ (blue line). Downcore percentage variations of *N. pachyderma* and of *U. peregrina* δ^18^O values (black lines, this study). Log (Ca/Ti) at IODP Site U1385, Iberian Margin^5^. Thick lines are 3-pt running averages. Horizontal dotted black lines indicate MIS boundaries from ref^4^. Horizontal grey boxes indicate stadial phases, progressively labeled, following the procedure by ref^6^. The black asterisk marks the position of the Parmenide ash layer.

Age/depth plot (black circles) with a polynomial fit regression. The red asterisk marks the position of the Parmenide ash layer, with its ^40^Ar/^39^Ar dating^1^.

| **Tie-point** | **Quota Blatta section (cm)** | **Age (ka) U1385** | **Sedimentation rate (cm/kyr)** |
| --- | --- | --- | --- |
|  |  |  |  |
| **7-IMS10** | 1870 | 670.26 |  |
|  |  |  | 24.7 |
| **7-IMS11** | 1710 | 676.74 |  |
|  |  |  | 53.7 |
| **7-IMS12** | 1490 | 680.84 |  |
|  |  |  | 37.4 |
| **7-IMS13** | 1370 | 684.05 |  |
|  |  |  | 76.1 |
| **7-IMS14** | 1160 | 686.81 |  |
|  |  |  | 32.4 |
| **7-IMS15** | 1090 | 688.97 |  |
|  |  |  | 42.2 |
| **7-IMS16** | 800 | 695.85 |  |
|  |  |  | 38.4 |
| **7-IMS17** | 520 | 703.15 |  |
|  |  |  | 33.2 |
| **7-IMS18** | 260 | 710.99 |  |
|  |  |  | 50.6 |
| **7-IMS19** | 130 | 713.56 |  |
|  |  |  | 73.0 |
| **8-IMS1** | -75 | 716.37 |  |

Tab. S1: the adopted tie-points from the Blatta section and the imported age from the IODP Site U1385^5^. The sedimentation rate ranges between 24.7 and 76.1 cm/kyr, averaging 42.2 cm/kyr. Resolution for the main sampling pace (10 cm) is between 131 and 405 yrs (Tab. 1), 237 yrs on average. The age/depth plot shows a polynomial fit with a correlation index of R^2^ = 0.988 (Fig. S2).

**Supplementary Material S3: Planktonic Foraminifera**

**
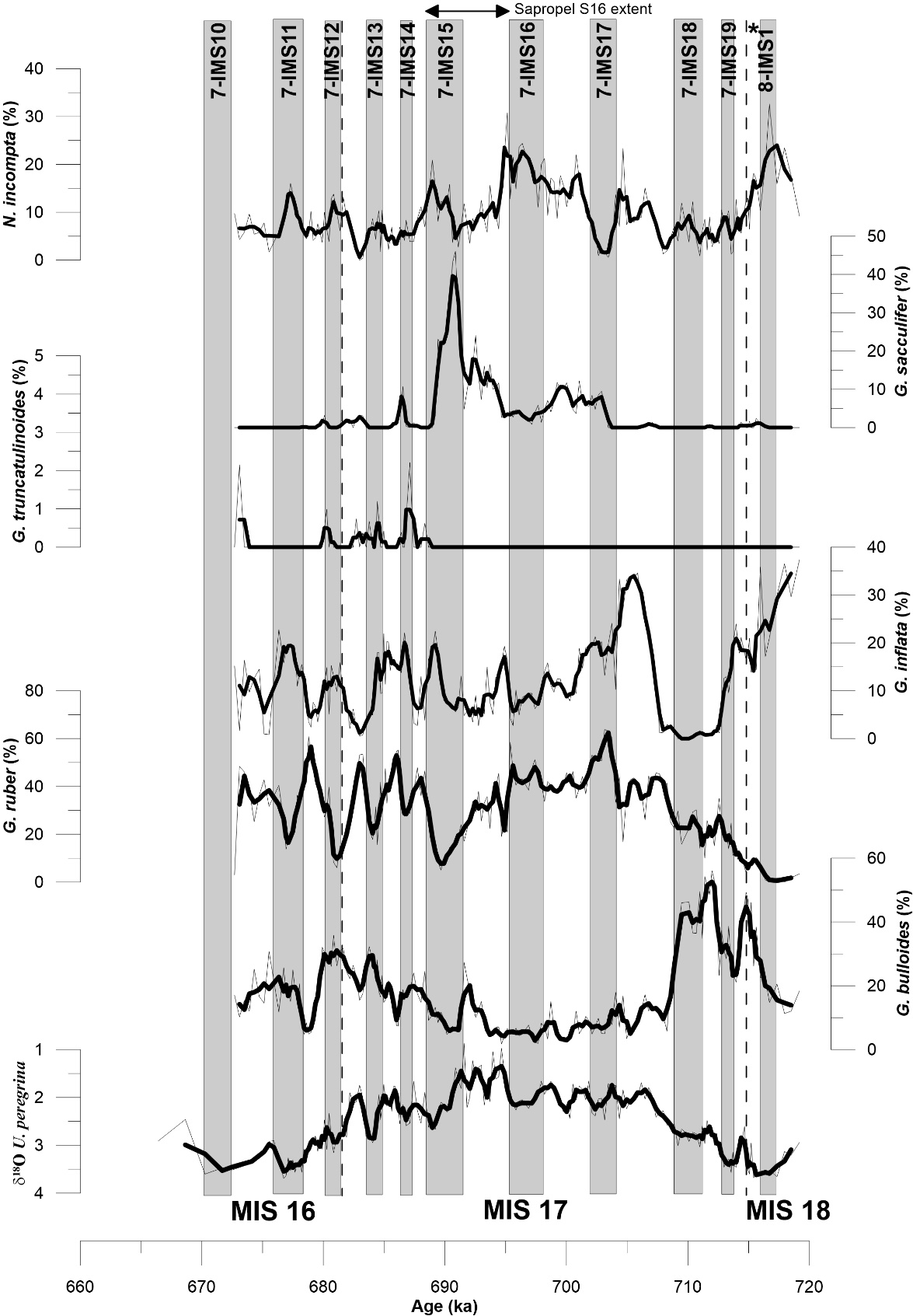
**

Fig. S3: downcore variations of selected planktonic foraminifera species at Blatta section, in comparison with *U. peregrina* oxygen isotopes variations. Thick lines are 3-pt running averages. Horizontal dotted black lines indicate MIS boundaries from^4^. Horizontal grey boxes indicate stadial phases, progressively labeled, following the procedure by ref^6^. The black asterisk marks the position of the Parmenide ash layer.

A total of 14 species were identified among planktonic foraminifera. *Globorotalia inflata* and *G. bulloides* dominate assemblages in the lower part of the record, across the MIS 18/MIS 17 transition. *Globigerinoides ruber* dominates the assemblages since ~ 705 ka (Fig. S3), though it exhibits large abundance fluctuations in the upper part of the record, with positive peaks associated with interstadials, alternated with abundance peaks of *Neogloboquadrina incompta* and *G. bulloides* in stadials (Fig. S3). *Globigerinoides sacculifer* is dominant in a narrow interval at the base of 7-IMS 15 stadial (Fig. S3). Ecological preferences of selected species are quoted in the main text of this manuscript. For the ecological preferences of other species indicated in Figure S3, readers should refer to reference^7^.

**Supplementary Material 4: Sapropel Isotopic Signature in shallow domains**

**
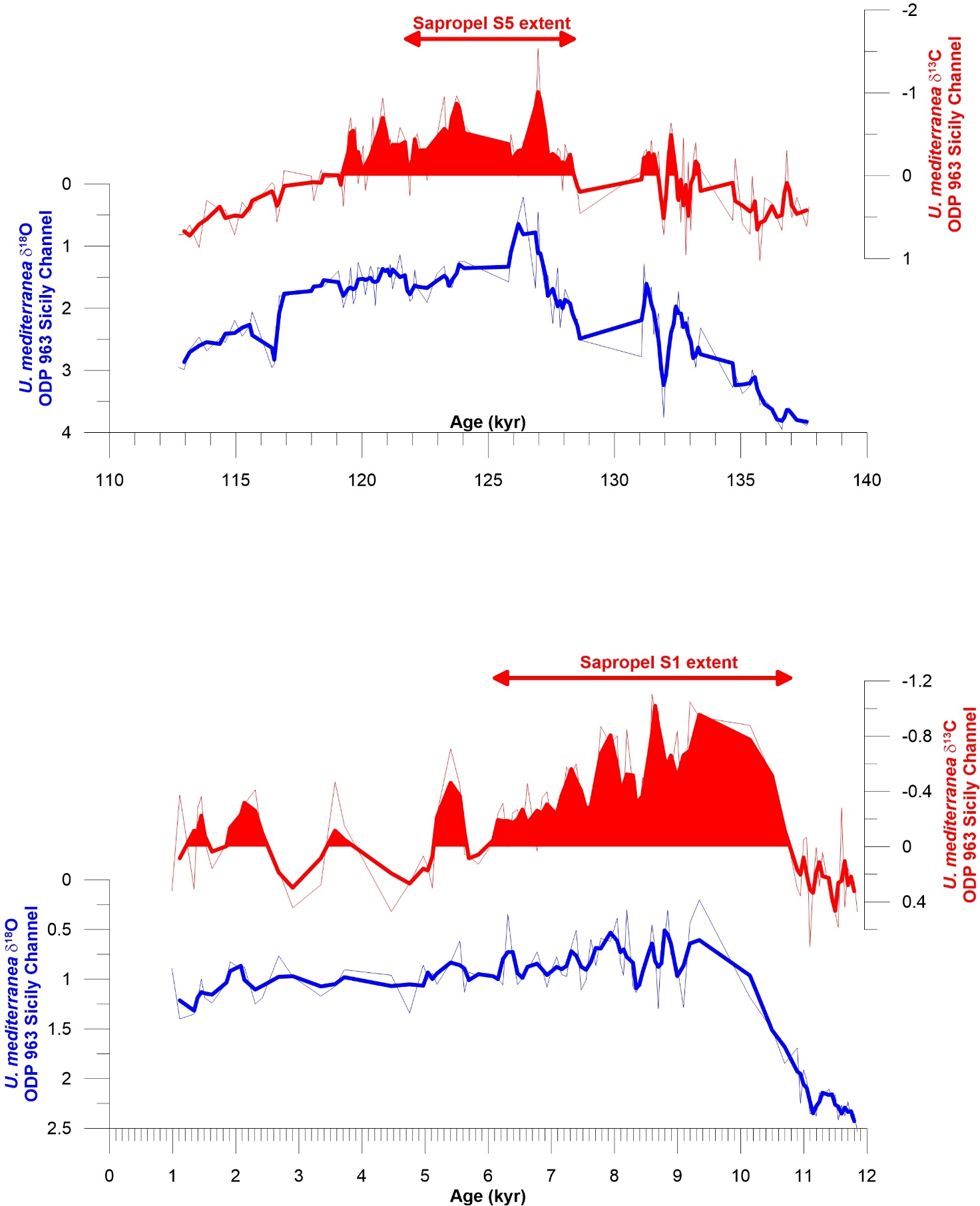
**

Figure S4: plot of *U. mediterranea* δ^18^O (blue line) and δ^13^C (red line) curves at ODP Site 963, Sicily Channel^8,9^, during the two most recent interglacials. The red filling indicates negative excursions in δ^13^C values. Thick lines are 3-pt running averages. Sapropel S1 and S5 timing follows ref^10^.

In the “type” area, i.e. the pelagic eastern Mediterranean domain, sapropel layers occur as thin horizons of blackish clays in the midst of a muddy succession^11^. In central Mediterranean open-marine hemipelagic settings (outer shelf to slope) characterized by high accumulation rates of muddy sediments, such as the Vrica section^12^, sapropel-equivalent layers are usually represented by very expanded and laminated bodies, locally rich in diatomitic horizons. In shallower contexts and/or depositional settings prone to the accumulation of coarser sediments, the relevant physical stratigraphic evidence may be flimsy, or even absent. Likewise, sapropel layers may be missing or partially missing in deep open-marine areas where sediment accumulation rates are especially low, because the reprise of ventilation following sapropel deposition may cause the burndown of the organic matter and the disappearance of any sedimentological evidence^13,14^. Either way, the geochemical signature of sapropels is likely to be preserved independently from the lithology. Tracers of increased productivity or thermohaline circulation perturbation are commonly employed in this regard, ranging from peaks in the relative concentration of Barium to the recognition of negative excursions within the carbon isotope records. The latter is the case of the Sicily Channel, the shallow sill that divides into two sub-basins the Mediterranean Sea, where sapropel were deposited up to the Early Pleistocene^11^, but the oceanographic perturbation associated with the zonal vertical circulation belt (surface and intermediate circulation cell) is clearly displayed by the negative δ^13^C excursion in benthic foraminifera shells, like in sapropel layers S1 and S5^8,9^ (Fig. S3).

**Supplementary Material 5: Benthic Foraminifera**

**
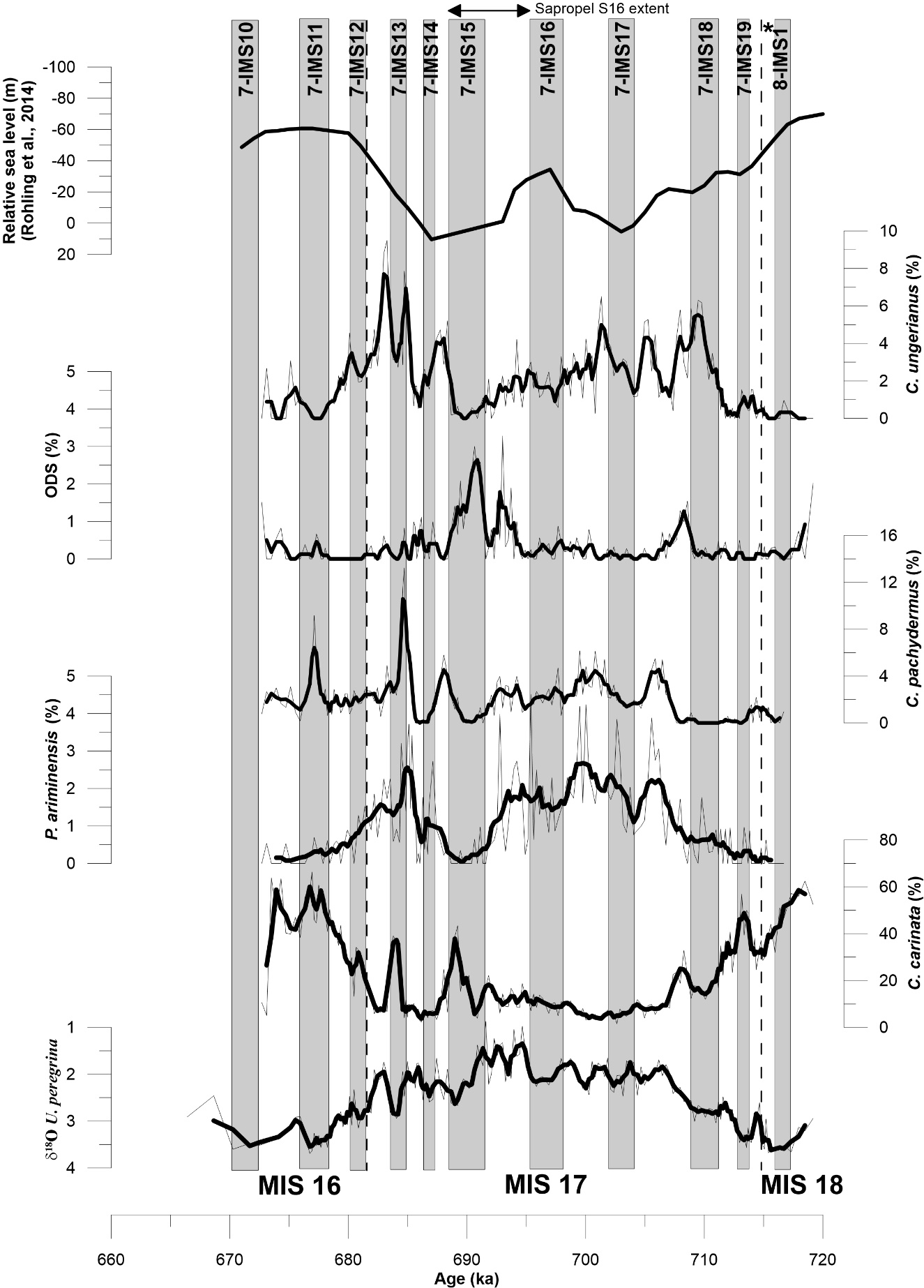
**

Figure S5: plot of selected benthic foraminifera taxa (%) at Blatta section and relative sea level variation from^15^. Thick lines in benthic foraminifera are 3-pt running averages. Horizontal dotted black lines indicate MIS boundaries from^4^ Horizontal grey boxes indicate stadial phases, progressively labeled, following the procedure by ref^6^. The timing extent of sapropel S16 follows^16^. The black asterisk marks the position of the Parmenide ash layer.

Benthic foraminifera assemblages are dominated by *Cassidulina carinata* during glacial MISs 18 and 16 (Fig. S5). The general good comparison with relative sea level suggests that *C. carinata* may benefit from supply of organic matter of continental origin^17^, while sea level is low (Fig. S5). The oxygen deficiency stress (ODS) species^18^ are especially abundant in narrow interval ~ 690 ka, where epibenthic species like *Cibicidoides pachydermus*, *Cibicidoides ungerianus* and *Planulina ariminensis* are absent or rare (Fig. S4). The P/B ratio for paleo-depth estimates envisage a 100-200 m bathymetry, which agrees with the outer shelf setting of the Blatta section.

**Supplementary Material S6: pollen flora and vegetational interpretation**

**Present-day vegetation in Southern Italy**

The mean annual temperature and precipitation in the Crotone area are ca. 15.6°C and 630 mm/year, respectively^19^. Winters are short, cool, and rainy (maximum precipitation rate: December, 110 mm/month), summers are long, warm, and dry (minimum precipitation rate: June, 9 mm/month).

The presence of high mountains surrounding the Crotone basin allows for as sharp organization of the vegetation into belts, as follows^20^:

a) Thermomediterranean Belt: prostrate shrubs, maquis, matorral and steppe-like elements (*Ephedra* and *Artemisia*) that thrive in arid coastal and lowland areas (total precipitation between 300 and 450 mm/yr).

b) Mesomediterranean Belt: shrubby maquis to closed evergreen forest dominated by *Quercus ilex* and other evergreen oaks, *Pistacia*, *Olea* and *Phyllirea*. In Southern Italy, this belt prospers where total precipitation is between 450 and 600 mm/yr and rainfalls mainly occur during the winter.

c) Supramediterranean Belt: mixed deciduous forest with *Quercus*, *Ulmus*, *Carpinus*, *Castanea* and others. It flourishes under a mild, seasonal-driven climate with a minimum rainfall of 600 mm/yr and mean annual temperatures above 10°C. In Southern Italy, distribution of this belt is limited by winter water availability and temperatures.

d) Southern Apennine Beech Woods: a ‘cold’ forest assemblage, dominated by *Abies* and/or *Fagus*, indicative of abundant, year-round precipitations, with wet summers and weak seasonal contrast. In the Calabria region, this assemblage develops above ca. 1500 m a.s.l., where the annual average temperature is below 10°C and precipitation is above 1700 mm/yr.

**The Blatta pollen record and its interpretation**

All the investigated samples contain well preserved pollen. Pollen assemblages are dominated by *Pinus* spp., a common scenario in marine sediments^21^ (Capraro et al., 2005). Following a well-established practice^21^, relative pollen abundances have been calculated excluding this taxon. Arboreal and non-arboreal plants (NAP) are present throughout. We have established eight ecologically significant vegetation groups that point to the present-day distribution of vegetation in Southern Italy, as described above. For each group, component pollen types are given in decreasing order of abundance, as follows (Figure S6).

ARBOREAL PLANTS

1) **Mediterranean xerophilous trees and shrubs**: includes *Quercus ilex/coccifera*, *Phyllirea*, *Pistacia*, *Olea*;

2) **Juglandaceae**: includes *Pterocarya*, *Carya* and *Juglans*, forest elements of the so-called “Tertiary” European flora;

3) **Temperate deciduous broadleaved trees**: includes deciduous-type *Quercus* pollen and small amounts of *Ulmus*, *Carpinus*, *Ostrya*, *Tilia*, *Castanea* and others;

4) **Tsuga + Cedrus** water-demanding conifers of the “Tertiary” European flora;

5) **Picea + Abies**: includes *Abies*, small amounts of *Picea* and sporadic grains of *Betula* and *Fagus*.

NON-ARBOREAL PLANTS:

6) **Steppe plants**: includes *Artemisia* and *Ephedra*;

7) **Halophilic plants**: includes Chenopodiaceae/Amaranthaceae and sporadic *Armeria* and *Limonium*;

8) **Ubiquitous herbs**: includes Asteraceae, Poaceae, Apiaceae, Ranunculaceae, etc.

To further simplify the paleoecological and paleoclimatic interpretation as discussed in the main article, groups have been clumped into supergroups suggestive of broad yet specific climatic conditions, as follows (Fig. S6).

- **Temperate broadleaved trees** (**groups 1+2+3**): pronounced seasonality with warm, dry summers and mild, cool winters; rainfall in the order of 600-700 mm/yr, mainly concentrated during the winter.
- **Mountain forest elements** (**groups 4+5**): temperate to cool/cold climates with abundant annual precipitations evenly distributed throughout the year.
- **Non arboreal plants** (groups **6+7+8**): indicative of dry to arid conditions throughout the year, hampering the persistence of a closed forest state.

**Fig. S6**: relative percentual abundances of ecological significant pollen groups presented above, calculated without *Pinus* spp. Red arrows indicate the stadial intervals (as found and labeled in the Iberian margin record) characterized by increased abundances of water-demanding forest elements (see text for details).

**Supplementary Material S7: multispecies planktonic and benthic foraminifera stable isotopes**

**
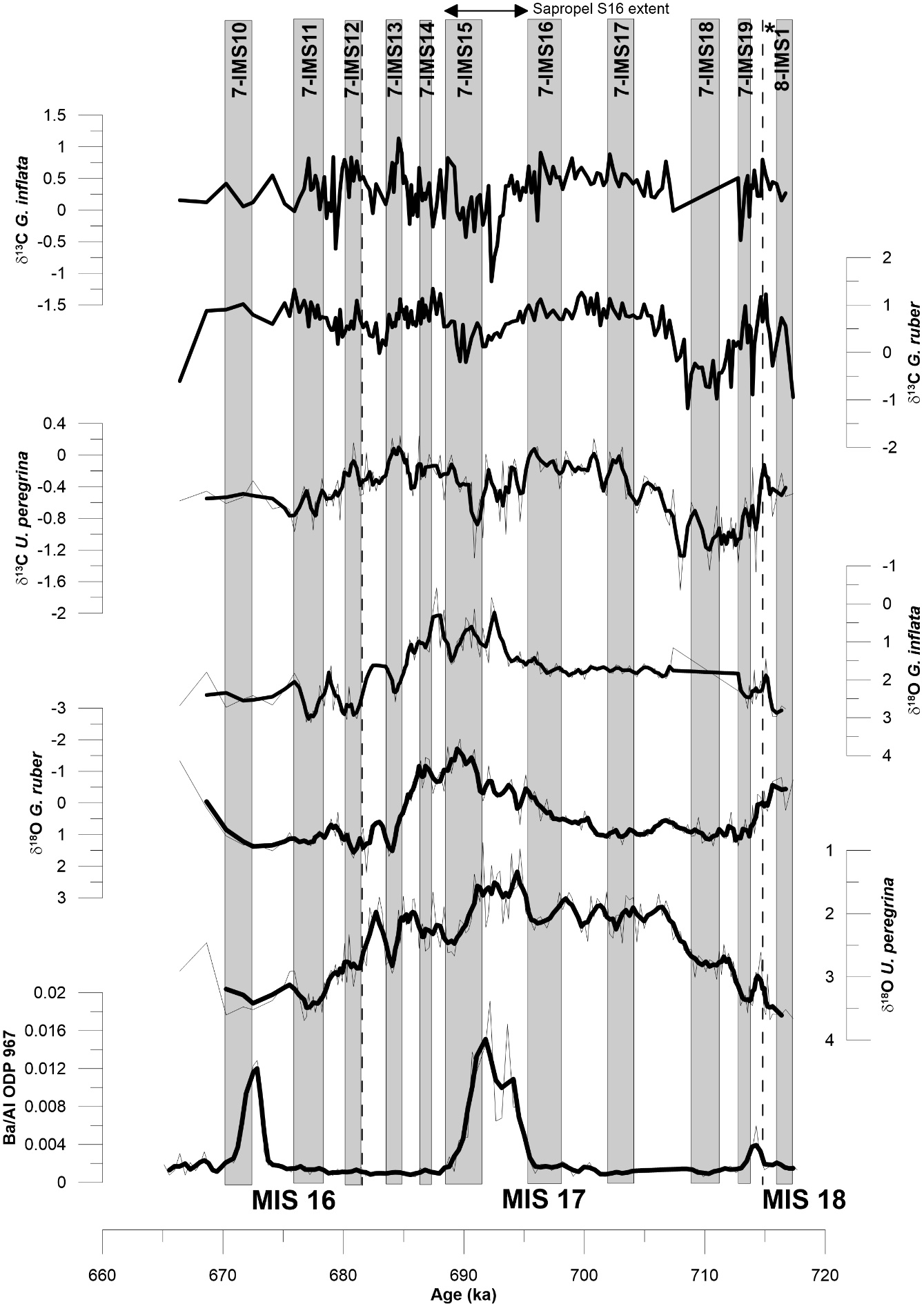
**

Fig. S7: downcore variations of planktonic (*G. ruber* and *G. inflata*) and benthic foraminifera (*U. peregrina*) stable isotopes at Blatta section, in comparison with Ba/Al values from the deep-sea eastern Mediterranean Sea^16^. Thick lines are 3-pt running averages. Horizontal dotted black lines indicate MIS boundaries from ref^4^. Horizontal grey boxes indicate stadial phases, progressively labeled, following the procedure by ref^6^ Martrat et al. (2007). The black asterisk marks the position of the Parmenide ash layer.

| **Quota (cm)** | **Age** | **δ^18^O** | **δ^13^C** | **δ^18^O** | **δ^13^C** |  | **Quota (cm)** | **Age** | **δ^18^O** | **δ^13^C** |
| --- | --- | --- | --- | --- | --- | --- | --- | --- | --- | --- |
| **Blatta** | **(kyr)** | ***U. peregrina*** | ***U. peregrina*** | ***G. ruber*** | ***G. ruber*** |  | **Blatta** | **(kyr)** | ***G. inflata*** | ***G. inflata*** |
| -280 | 719.18 | 2.94 | -11.25 | -1.19 | -1.05 |  | -280 | 719.18 | 2.92 | 0.34 |
| -230 | 718.49 | 3.21 | -1.18 | -0.67 | 1.16 |  | -230 | 718.49 | 2.61 | 0.27 |
| -190 | 717.95 | 3.13 | -8.68 | -0.88 | -0.76 |  | -145 | 717.33 | 2.76 | 0.43 |
| -145 | 717.33 | 3.66 | -0.49 | -0.73 | -0.94 |  | -100 | 716.71 | 2.77 | 0.27 |
| -100 | 716.71 | 3.52 | -0.52 | 0.23 | 0.56 |  | -75 | 716.37 | 2.69 | 0.15 |
| -75 | 716.37 | 3.59 | -0.23 | -0.81 | 0.73 |  | -45 | 715.96 | 2.97 | 0.42 |
| -20 | 715.62 | 3.59 | -0.78 | -0.68 | -0.28 |  | -20 | 715.62 | 2.97 | 0.44 |
| -5 | 715.41 | 3.68 | -0.27 | -0.20 | 0.36 |  | -5 | 715.41 | 2.41 | 0.32 |
| 10 | 715.20 | 2.96 | -0.44 | 0.26 | 0.63 |  | 10 | 715.20 | 2.00 | 0.47 |
| 20 | 715.07 | 3.60 | -0.13 | 0.17 | 1.23 |  | 20 | 715.07 | 1.44 | 0.55 |
| 30 | 714.93 | 3.52 | -0.21 | -0.39 | 0.77 |  | 30 | 714.93 | 2.21 | 0.66 |
| 40 | 714.79 | 3.22 | -0.04 | 0.51 | 0.65 |  | 40 | 714.79 | 2.48 | 0.80 |
| 50 | 714.66 | 2.61 | -0.46 | -0.17 | 1.17 |  | 50 | 714.66 | 2.03 | 0.22 |
| 70 | 714.38 | 2.99 | -0.50 | -0.28 | 0.65 |  | 70 | 714.38 | 2.40 | 0.22 |
| 80 | 714.25 | 2.93 | -1.48 | 0.61 | 0.41 |  | 80 | 714.25 | 2.52 | 0.43 |
| 90 | 714.11 | 3.18 | -0.84 | 0.98 | 0.15 |  | 90 | 714.11 | 1.74 | 0.63 |
| 100 | 713.97 | 3.46 | -0.16 | 0.07 | -0.89 |  | 100 | 713.97 | 2.64 | 0.34 |
| 110 | 713.83 | 3.43 | -0.92 | 1.04 | 0.49 |  | 110 | 713.83 | 2.49 | 0.39 |
| 120 | 713.70 | 3.14 | -1.06 | 1.10 | 0.57 |  | 120 | 713.70 | 2.14 | -0.11 |
| 130 | 713.56 | 3.57 | -0.69 | 1.03 | 0.21 |  | 130 | 713.56 | 2.77 | 0.54 |
| 140 | 713.36 | 3.29 | -0.54 | 0.51 | 0.98 |  | 140 | 713.36 | 2.48 | 0.38 |
| 150 | 713.16 | 3.35 | -0.82 | 1.01 | 0.60 |  | 160 | 712.97 | 2.06 | -0.48 |
| 160 | 712.97 | 3.46 | -0.98 | 0.93 | 0.08 |  | 170 | 712.77 | 2.29 | 0.51 |
| 170 | 712.77 | 3.02 | -1.37 | 1.38 | 0.22 |  | 380 | 707.37 | 1.16 | -0.01 |
| 180 | 712.57 | 3.13 | -0.86 | 0.91 | 0.14 |  | 390 | 707.07 | 1.83 | 0.42 |
| 190 | 712.37 | 2.75 | -1.20 | 1.26 | 0.23 |  | 400 | 706.77 | 1.97 | 0.77 |
| 200 | 712.18 | 3.00 | -0.79 | 0.29 | -0.73 |  | 410 | 706.47 | 1.83 | 0.34 |
| 210 | 711.98 | 2.62 | -1.13 | 0.79 | 0.25 |  | 420 | 706.17 | 1.95 | 0.29 |
| 220 | 711.78 | 2.73 | -1.01 | 1.12 | -0.10 |  | 430 | 705.86 | 1.71 | 0.20 |
| 230 | 711.58 | 2.48 | -1.24 | 1.11 | -0.33 |  | 440 | 705.56 | 1.74 | 0.21 |
| 240 | 711.39 | 2.96 | -0.78 | 0.82 | -0.43 |  | 450 | 705.26 | 1.72 | 0.60 |
| 250 | 711.19 | 2.59 | -1.35 | 0.82 | -0.43 |  | 460 | 704.96 | 1.58 | 0.22 |
| 260 | 710.99 | 3.04 | -0.64 | 0.86 | -0.98 |  | 470 | 704.66 | 1.73 | 0.33 |
| 270 | 710.69 | 2.76 | -1.06 | 1.36 | -0.17 |  | 480 | 704.36 | 1.93 | 0.48 |
| 280 | 710.39 | 2.73 | -1.46 | 0.92 | -0.73 |  | 490 | 704.05 | 1.84 | 0.42 |
| 290 | 710.09 | 2.92 | -1.08 | 1.16 | -0.72 |  | 500 | 703.75 | 1.63 | 0.49 |
| 300 | 709.78 | 2.73 | -0.96 | 1.27 | -0.31 |  | 510 | 703.45 | 1.77 | 0.56 |
| 310 | 709.48 | 2.66 | -0.79 | 0.45 | -0.33 |  | 520 | 703.15 | 1.85 | 0.57 |
| 320 | 709.18 | 3.01 | -0.73 | 1.03 | -0.23 |  | 530 | 702.89 | 1.63 | 0.37 |
| 330 | 708.88 | 2.56 | -0.60 | 1.05 | -0.46 |  | 540 | 702.63 | 1.61 | 0.48 |
| 340 | 708.58 | 2.57 | -1.02 | 0.68 | -1.18 |  | 550 | 702.37 | 1.83 | 0.66 |
| 350 | 708.28 | 2.58 | -1.09 | 0.95 | 0.27 |  | 560 | 702.11 | 1.80 | 0.88 |
| 360 | 707.97 | 2.29 | -1.71 | 0.82 | -0.08 |  | 570 | 701.85 | 1.70 | 0.47 |
| 370 | 707.67 | 2.30 | -1.01 | 0.62 | -0.17 |  | 580 | 701.59 | 1.63 | 0.39 |
| 380 | 707.37 | 2.41 | -0.43 | 0.81 | 0.28 |  | 590 | 701.33 | 1.67 | 0.57 |
| 390 | 707.07 | 1.97 | -0.89 | 0.46 | 0.67 |  | 600 | 701.06 | 1.78 | 0.59 |
| 400 | 706.77 | 1.79 | -0.79 | 0.55 | 0.31 |  | 610 | 700.80 | 1.70 | 0.43 |
| 410 | 706.47 | 2.04 | -0.45 | 0.63 | 0.98 |  | 620 | 700.54 | 1.59 | 0.41 |
| 420 | 706.17 | 1.69 | -0.45 | 0.56 | 0.53 |  | 630 | 700.28 | 1.71 | 0.51 |
| 430 | 705.86 | 1.99 | -0.35 | 0.90 | 0.36 |  | 640 | 700.02 | 2.00 | 0.59 |
| 440 | 705.56 | 2.02 | -0.49 | 0.80 | 0.77 |  | 650 | 699.76 | 1.71 | 0.67 |
| 450 | 705.26 | 2.05 | -0.30 | 1.05 | 0.78 |  | 660 | 699.50 | 1.80 | 0.61 |
| 460 | 704.96 | 2.24 | -0.26 | 0.85 | 1.01 |  | 670 | 699.24 | 1.70 | 0.50 |
| 470 | 704.66 | 1.84 | -0.60 | 1.08 | 0.74 |  | 680 | 698.98 | 1.94 | 0.82 |
| 480 | 704.36 | 2.45 | -0.74 | 1.05 | 0.72 |  | 690 | 698.72 | 1.88 | 0.65 |
| 490 | 704.05 | 1.74 | -0.53 | 0.84 | 0.79 |  | 700 | 698.46 | 1.85 | 0.49 |
| 500 | 703.75 | 1.78 | -0.37 | 0.77 | 0.75 |  | 710 | 698.20 | 1.68 | 0.33 |
| 510 | 703.45 | 1.71 | -0.56 | 0.91 | 0.77 |  | 720 | 697.94 | 1.80 | 0.52 |
| 520 | 703.15 | 2.47 | 0.10 | 1.32 | 1.04 |  | 730 | 697.68 | 1.57 | 0.51 |
| 530 | 702.89 | 2.02 | -0.06 | 0.96 | 0.71 |  | 740 | 697.41 | 1.76 | 0.68 |
| 540 | 702.63 | 2.03 | -0.08 | 0.87 | 0.82 |  | 750 | 697.15 | 1.92 | 0.42 |
| 550 | 702.37 | 2.39 | -0.14 | 0.75 | 0.93 |  | 760 | 696.89 | 1.69 | 0.57 |
| 560 | 702.11 | 2.32 | 0.04 | 1.13 | 1.13 |  | 780 | 696.37 | 1.86 | 0.91 |
| 570 | 701.85 | 1.65 | -0.52 | 0.98 | 0.73 |  | 790 | 696.11 | 1.24 | -0.17 |
| 580 | 701.59 | 2.05 | -0.42 | 1.06 | 0.92 |  | 800 | 695.85 | 1.62 | 0.60 |
| 590 | 701.33 | 1.83 | -0.42 | 1.03 | 0.57 |  | 810 | 695.61 | 1.41 | 0.28 |
| 600 | 701.06 | 1.76 | 0.00 | 0.94 | 1.24 |  | 820 | 695.38 | 1.53 | 0.69 |
| 610 | 700.80 | 2.05 | 0.21 | 1.01 | 0.80 |  | 830 | 695.14 | 1.59 | 0.28 |
| 620 | 700.54 | 1.72 | -0.15 | 0.63 | 1.15 |  | 840 | 694.90 | 1.71 | 0.35 |
| 630 | 700.28 | 2.44 | -0.19 | 0.55 | 0.81 |  | 850 | 694.66 | 1.26 | 0.16 |
| 640 | 700.02 | 2.15 | -0.25 | 0.25 | 1.16 |  | 860 | 694.43 | 1.55 | 0.45 |
| 650 | 699.76 | 2.30 | -0.11 | 0.86 | 1.26 |  | 870 | 694.19 | 1.42 | 0.26 |
| 660 | 699.50 | 2.12 | -0.19 | 0.54 | 1.16 |  | 880 | 693.95 | 1.48 | 0.55 |
| 670 | 699.24 | 1.98 | -0.16 | 0.53 | 0.89 |  | 890 | 693.71 | 1.66 | 0.38 |
| 680 | 698.98 | 1.75 | -0.27 | 0.70 | 0.71 |  | 900 | 693.48 | 1.12 | 0.38 |
| 690 | 698.72 | 1.66 | -0.06 | 0.65 | 0.84 |  | 910 | 693.24 | 1.01 | -0.08 |
| 700 | 698.46 | 1.80 | 0.05 | 0.40 | 0.91 |  | 920 | 693.00 | 0.86 | -0.11 |
| 710 | 698.20 | 1.93 | -0.24 | 0.56 | 0.70 |  | 930 | 692.77 | 0.72 | -0.55 |
| 720 | 697.94 | 1.66 | -0.43 | 0.54 | 0.70 |  | 940 | 692.53 | 0.14 | -0.74 |
| 730 | 697.68 | 1.94 | -0.04 | 0.49 | 0.79 |  | 950 | 692.29 | -0.17 | -1.13 |
| 740 | 697.41 | 2.05 | -0.09 | 0.47 | 0.89 |  | 960 | 692.05 | 1.56 | 0.38 |
| 750 | 697.15 | 2.09 | -0.10 | 0.52 | 0.83 |  | 970 | 691.82 | 1.21 | -0.03 |
| 760 | 696.89 | 2.23 | -0.10 | -0.13 | 0.74 |  | 980 | 691.58 | 0.63 | -0.23 |
| 780 | 696.37 | 2.05 | -0.21 | 0.57 | 1.12 |  | 990 | 691.34 | 1.41 | 0.24 |
| 790 | 696.11 | 2.08 | 0.11 | -0.12 | 1.02 |  | 1000 | 691.11 | 1.07 | 0.19 |
| 800 | 695.85 | 2.26 | 0.06 | -0.38 | 0.68 |  | 1010 | 690.87 | 0.14 | -0.34 |
| 810 | 695.61 | 2.14 | 0.07 | -0.03 | 0.86 |  | 1020 | 690.63 | 1.17 | 0.21 |
| 820 | 695.38 | 2.02 | -0.01 | -0.13 | 1.00 |  | 1030 | 690.39 | 0.49 | 0.00 |
| 830 | 695.14 | 1.99 | -0.16 | -0.90 | 0.88 |  | 1040 | 690.16 | 0.36 | -0.43 |
| 840 | 694.90 | 1.49 | -0.42 | -0.33 | 0.63 |  | 1050 | 689.92 | 1.24 | 0.03 |
| 850 | 694.66 | 0.97 | -0.84 | 0.69 | 0.86 |  | 1060 | 689.68 | 0.89 | -0.15 |
| 860 | 694.43 | 1.57 | -0.20 | -0.09 | 0.67 |  | 1070 | 689.44 | 0.99 | -0.03 |
| 870 | 694.19 | 1.61 | -0.05 | -0.07 | 0.66 |  | 1080 | 689.21 | 1.53 | 0.68 |
| 880 | 693.95 | 1.05 | -0.28 | -0.75 | 0.64 |  | 1090 | 688.97 | 1.60 | 0.74 |
| 890 | 693.71 | 2.09 | -0.13 | -0.59 | 0.61 |  | 1100 | 688.66 | 1.40 | 0.82 |
| 900 | 693.48 | 1.75 | -1.01 | -0.36 | 0.60 |  | 1110 | 688.35 | 0.16 | -0.12 |
| 910 | 693.24 | 2.16 | -0.07 | -0.35 | 0.48 |  | 1120 | 688.04 | 1.17 | 0.63 |
| 920 | 693.00 | 1.33 | -0.84 | -0.25 | 0.32 |  | 1130 | 687.74 | -0.42 | 0.08 |
| 930 | 692.77 | 1.37 | -0.43 | -0.16 | 0.41 |  | 1140 | 687.43 | 0.20 | -0.26 |
| 940 | 692.53 | 1.61 | -0.47 | -0.35 | 0.38 |  | 1150 | 687.12 | 1.28 | 0.43 |
| 950 | 692.29 | 1.23 | -0.36 | -0.53 | 0.26 |  | 1160 | 686.81 | 1.17 | 0.17 |
| 960 | 692.05 | 2.00 | -0.21 | -1.18 | 0.35 |  | 1170 | 686.68 | 1.03 | 0.26 |
| 970 | 691.82 | 2.22 | 0.01 | -0.35 | 0.13 |  | 1180 | 686.55 | 1.00 | 0.15 |
| 980 | 691.58 | 0.87 | -1.05 | -0.06 | 0.12 |  | 1190 | 686.42 | 1.07 | 0.36 |
| 990 | 691.34 | 1.82 | -0.56 | -0.53 | 0.51 |  | 1200 | 686.28 | 1.04 | 0.67 |
| 1000 | 691.11 | 1.65 | -0.78 | -0.55 | 0.62 |  | 1210 | 686.15 | 0.86 | -0.10 |
| 1010 | 690.87 | 1.38 | -1.30 | -1.69 | 0.46 |  | 1220 | 686.02 | 1.23 | 0.25 |
| 1020 | 690.63 | 2.09 | -0.34 | -1.37 | 0.31 |  | 1230 | 685.89 | 1.49 | 0.03 |
| 1030 | 690.39 | 2.26 | -0.50 | -1.25 | 0.04 |  | 1240 | 685.76 | 1.18 | -0.08 |
| 1040 | 690.16 | 2.31 | -0.27 | -1.32 | -0.20 |  | 1250 | 685.63 | 1.10 | 0.40 |
| 1050 | 689.92 | 2.15 | -0.35 | -1.12 | 0.59 |  | 1260 | 685.50 | 0.94 | -0.22 |
| 1060 | 689.68 | 2.10 | -0.47 | -2.02 | -0.19 |  | 1270 | 685.36 | 1.41 | 0.20 |
| 1070 | 689.44 | 2.37 | -0.35 | -1.68 | 0.12 |  | 1280 | 685.23 | 1.57 | 0.45 |
| 1080 | 689.21 | 2.67 | -0.21 | -1.43 | 0.65 |  | 1290 | 685.10 | 1.85 | 0.53 |
| 1090 | 688.97 | 2.60 | 0.00 | -0.93 | 0.66 |  | 1300 | 684.97 | 1.66 | 0.42 |
| 1100 | 688.66 | 2.62 | -0.14 | -1.78 | 0.55 |  | 1310 | 684.84 | 2.03 | 0.90 |
| 1110 | 688.35 | 1.89 | -0.57 | -0.37 | 0.54 |  | 1320 | 684.71 | 2.12 | 1.02 |
| 1120 | 688.04 | 2.54 | 0.00 | -0.99 | 1.24 |  | 1330 | 684.58 | 2.07 | 1.13 |
| 1130 | 687.74 | 2.28 | -0.25 | -0.72 | 0.81 |  | 1340 | 684.44 | 2.20 | 0.75 |
| 1140 | 687.43 | 1.66 | -0.16 | -0.30 | 1.34 |  | 1350 | 684.31 | 2.60 | 0.72 |
| 1150 | 687.12 | 2.54 | 0.01 | -1.38 | 0.66 |  | 1360 | 684.18 | 2.21 | 0.28 |
| 1160 | 686.81 | 2.59 | -0.29 | -1.44 | 0.99 |  | 1370 | 684.05 | 2.15 | 0.75 |
| 1170 | 686.68 | 2.28 | -0.18 | -0.68 | 0.74 |  | 1380 | 683.78 | 1.70 | 0.31 |
| 1180 | 686.55 | 2.16 | -0.27 | -0.47 | 1.13 |  | 1390 | 683.52 | 1.64 | 0.09 |
| 1190 | 686.42 | 2.25 | -0.32 | -1.34 | 0.60 |  | 1420 | 682.71 | 1.61 | 0.41 |
| 1200 | 686.28 | 2.55 | 0.24 | -0.97 | 1.06 |  | 1430 | 682.45 | 1.60 | -0.05 |
| 1210 | 686.15 | 2.08 | -0.26 | -1.19 | 0.75 |  | 1440 | 682.18 | 1.66 | 0.32 |
| 1220 | 686.02 | 2.03 | -0.39 | -0.48 | 0.56 |  | 1450 | 681.91 | 2.04 | 0.40 |
| 1230 | 685.89 | 1.76 | -0.33 | -0.53 | 0.64 |  | 1460 | 681.64 | 2.21 | 0.53 |
| 1240 | 685.76 | 1.77 | -0.52 | -0.68 | 0.59 |  | 1470 | 681.38 | 2.66 | 0.48 |
| 1250 | 685.63 | 2.30 | -0.34 | -0.05 | 0.87 |  | 1480 | 681.11 | 3.03 | 0.77 |
| 1260 | 685.50 | 2.02 | -0.42 | -0.33 | 0.74 |  | 1490 | 680.84 | 2.88 | 0.46 |
| 1270 | 685.36 | 2.13 | 0.03 | -0.15 | 0.53 |  | 1500 | 680.65 | 2.89 | 0.83 |
| 1280 | 685.23 | 2.18 | -0.25 | -0.32 | 0.96 |  | 1510 | 680.47 | 2.18 | 0.20 |
| 1290 | 685.10 | 2.02 | 0.04 | 0.38 | 0.70 |  | 1520 | 680.28 | 3.05 | 0.65 |
| 1300 | 684.97 | 1.73 | -0.08 | 0.27 | 0.91 |  | 1530 | 680.09 | 2.75 | 0.80 |
| 1310 | 684.84 | 2.14 | -0.05 | 0.17 | 0.86 |  | 1540 | 679.91 | 2.93 | 0.66 |
| 1320 | 684.71 | 2.47 | 0.25 | 0.66 | 1.12 |  | 1550 | 679.72 | 2.82 | 0.32 |
| 1330 | 684.58 | 1.94 | 0.02 | 0.47 | 0.71 |  | 1560 | 679.54 | 2.10 | -0.15 |
| 1340 | 684.44 | 2.28 | 0.03 | 0.72 | 0.82 |  | 1570 | 679.35 | 2.38 | -0.61 |
| 1350 | 684.31 | 2.75 | -0.02 | 1.09 | 0.55 |  | 1580 | 679.16 | 2.81 | 0.84 |
| 1360 | 684.18 | 2.87 | 0.05 | 1.40 | 0.63 |  | 1590 | 678.98 | 1.63 | -0.02 |
| 1370 | 684.05 | 2.94 | 0.22 | 1.45 | 0.53 |  | 1600 | 678.79 | 1.89 | 0.11 |
| 1380 | 683.78 | 2.75 | -0.19 | 1.68 | 0.80 |  | 1610 | 678.60 | 1.90 | -0.13 |
| 1390 | 683.52 | 2.82 | -0.24 | 1.05 | 0.15 |  | 1620 | 678.42 | 2.70 | 0.53 |
| 1400 | 683.25 | 2.01 | -0.35 | 0.60 | 0.26 |  | 1630 | 678.23 | 2.49 | 0.26 |
| 1410 | 682.98 | 1.74 | -0.26 | 0.54 | -0.01 |  | 1640 | 678.04 | 2.15 | 0.01 |
| 1420 | 682.71 | 2.09 | -0.46 | 0.65 | 0.62 |  | 1650 | 677.86 | 2.85 | 0.41 |
| 1430 | 682.45 | 2.13 | -0.21 | 0.72 | 0.33 |  | 1660 | 677.67 | 2.96 | 0.54 |
| 1440 | 682.18 | 1.90 | -0.46 | 0.85 | 0.36 |  | 1670 | 677.49 | 2.82 | 0.35 |
| 1450 | 681.91 | 2.83 | 0.03 | 2.21 | 0.63 |  | 1680 | 677.30 | 3.13 | 0.31 |
| 1460 | 681.64 | 2.68 | -0.49 | 0.81 | 0.53 |  | 1690 | 677.11 | 2.85 | 0.82 |
| 1470 | 681.38 | 2.83 | -0.51 | 1.22 | 0.70 |  | 1700 | 676.93 | 3.12 | 0.57 |
| 1480 | 681.11 | 2.82 | -0.14 | 1.32 | 1.09 |  | 1710 | 676.74 | 2.73 | 0.34 |
| 1490 | 680.84 | 3.15 | 0.15 | 1.74 | 0.50 |  | 1720 | 676.34 | 2.07 | 0.18 |
| 1500 | 680.65 | 2.83 | -0.24 | 1.61 | 0.66 |  | 1730 | 675.93 | 1.83 | -0.02 |
| 1510 | 680.47 | 2.33 | -0.27 | 0.92 | 0.49 |  | 1750 | 675.12 | 2.26 | 0.10 |
| 1520 | 680.28 | 3.09 | -0.27 | 1.24 | 0.55 |  | 1775 | 674.11 | 2.67 | 0.55 |
| 1530 | 680.09 | 2.47 | -0.16 | 0.90 | 0.40 |  | 1815 | 672.49 | 2.42 | 0.12 |
| 1540 | 679.91 | 3.08 | -0.23 | 1.35 | 0.33 |  | 1835 | 671.68 | 2.51 | 0.06 |
| 1550 | 679.72 | 2.87 | -0.82 | 1.13 | 0.90 |  | 1870 | 670.26 | 2.73 | 0.42 |
| 1560 | 679.54 | 3.13 | -0.32 | 0.29 | 0.18 |  | 1910 | 668.64 | 1.80 | 0.12 |
| 1570 | 679.35 | 2.80 | -0.40 | 1.06 | 0.73 |  | 1965 | 666.41 | 2.68 | 0.15 |
| 1580 | 679.16 | 2.95 | -0.65 | 0.81 | 0.48 |  |  |  |  |  |
| 1590 | 678.98 | 2.89 | -0.50 | 0.50 | 0.47 |  |  |  |  |  |
| 1600 | 678.79 | 2.97 | -0.49 | 0.76 | 0.56 |  |  |  |  |  |
| 1610 | 678.60 | 3.36 | -0.65 | 1.10 | 0.73 |  |  |  |  |  |
| 1620 | 678.42 | 3.09 | -0.53 | 1.05 | 0.73 |  |  |  |  |  |
| 1630 | 678.23 | 3.56 | -0.29 | 1.12 | 0.75 |  |  |  |  |  |
| 1640 | 678.04 | 3.30 | -0.52 | 0.88 | 0.70 |  |  |  |  |  |
| 1650 | 677.86 | 3.36 | -0.77 | 0.92 | 0.99 |  |  |  |  |  |
| 1660 | 677.67 | 3.57 | -0.58 | 1.28 | 0.73 |  |  |  |  |  |
| 1670 | 677.49 | 3.31 | -0.95 | 1.04 | 0.93 |  |  |  |  |  |
| 1680 | 677.30 | 3.53 | -0.64 | 1.36 | 1.06 |  |  |  |  |  |
| 1690 | 677.11 | 3.26 | -0.43 | 1.29 | 0.55 |  |  |  |  |  |
| 1700 | 676.93 | 3.63 | -0.54 | 1.14 | 0.93 |  |  |  |  |  |
| 1710 | 676.74 | 3.69 | -0.39 | 1.26 | 1.09 |  |  |  |  |  |
| 1720 | 676.34 | 3.31 | -0.62 | 1.18 | 0.85 |  |  |  |  |  |
| 1730 | 675.93 | 2.91 | -0.97 | 1.25 | 1.33 |  |  |  |  |  |
| 1740 | 675.53 | 2.92 | -0.69 | 0.93 | 0.77 |  |  |  |  |  |
| 1750 | 675.12 | 3.12 | -0.64 | 1.21 | 1.04 |  |  |  |  |  |
| 1775 | 674.11 | 3.37 | -0.68 | 1.51 | 0.60 |  |  |  |  |  |
| 1815 | 672.49 | 3.52 | -0.32 | 1.29 | 0.79 |  |  |  |  |  |
| 1835 | 671.68 | 3.47 | -0.53 | 1.32 | 1.02 |  |  |  |  |  |
| 1870 | 670.26 | 3.60 | -0.61 | 1.04 | 0.90 |  |  |  |  |  |
| 1910 | 668.64 | 2.46 | -0.46 | 0.16 | 0.88 |  |  |  |  |  |
| 1965 | 666.41 | 2.91 | -0.58 | -1.33 | -0.60 |  |  |  |  |  |

Table S2: planktonic and benthic foraminifera stable isotope raw data in the Blatta section.

**Bibliography**

1. Capraro, L. *et al.* Chronology of the Lower-Middle Pleistocene succession of the south-western part of the Crotone Basin (Calabria, Southern Italy). *Quat Sci Rev* **30**, 1185–1200 (2011).

2. Massari, F., Prosser, G., Capraro, L., Fornaciari, E. & Consolaro, C. A revision of the stratigraphy and geology of the south-western part of the Neogene Crotone Basin (South Italy). *Italian Journal of Geosciences* **129**, 353–384 (2010).

3. Lourens, L. J. Revised tuning of Ocean Drilling Program Site 964 and KC01B (Mediterranean) and implications for the $δ$18O, tephra, calcareous nannofossil, and geomagnetic reversal chronologies of the past 1.1 Myr. *Paleoceanography* **19**, 1–20 (2004).

4. Lisiecki, L. E. & Raymo, M. E. A Pliocene-Pleistocene stack of 57 globally distributed benthic ?? 18O records. *Paleoceanography* **20**, 1–17 (2005).

5. Hodell, D. *et al.* A reference time scale for Site U1385 (Shackleton Site) on the SW Iberian Margin. *Glob Planet Change* **133**, 49–64 (2015).

6. Martrat, B. *et al.* Four climate cycles of recurring deep and surface water destabilizations on the Iberian margin. *Science* **317**, 502–507 (2007).

7. Schiebel, R. & Hemleben, C. *Planktic Foraminifers in the Modern Ocean*. (2017).

8. Incarbona, A., Sprovieri, M., Lirer, F. & Sprovieri, R. Surface and deep water conditions in the Sicily channel (central Mediterranean) at the time of sapropel S5 deposition. *Palaeogeogr Palaeoclimatol Palaeoecol* **306**, 243–248 (2011).

9. Incarbona, A. & Sprovieri, M. The Postglacial Isotopic Record of Intermediate Water Connects Mediterranean Sapropels and Organic-Rich Layers. *Paleoceanogr Paleoclimatol* **35**, e2020PA004009 (2020).

10. Grant, K. M. *et al.* The timing of Mediterranean sapropel deposition relative to insolation, sea-level and African monsoon changes. *Quat Sci Rev* **140**, 125–141 (2016).

11. Emeis, K.-C., Robertson, A. H. F., Richter, C. & Party, S. Site 967. In: Proceedings of the Ocean Drilling Program, Initial Reports, Leg 160 (Emeis, K.-C., Robertson, A.H.F. and Richter, C. Eds). in vol. 160 215–287 (1996).

12. Aguirre, E. & Pasini, G. The Pliocene-Pleistocence Boundary. *International Union of Geological Sciences* **8**, 116–120 (1985).

13. Thomson, J., Mercone, D., de Lange, G. J. & van Santvoort, P. J. M. Review of recent advances in the interpretation of eastern Mediterranean sapropel S1 from geochemical evidence. *Mar Geol* **153**, 77–89 (1999).

14. de Lange, G. J. *et al.* Synchronous basin-wide formation and redox-controlled preservation of a Mediterranean sapropel. *Nat Geosci* **1**, 606–610 (2008).

15. Rohling, E. J. *et al.* Sea-level and deep-sea-temperature variability over the past 5.3 million years. *Nature* **508**, 477–482 (2014).

16. Grant, K. M. *et al.* A 3 million year index for North African humidity/aridity and the implication of potential pan-African Humid periods. *Quat Sci Rev* **171**, 100–118 (2017).

17. Mojtahid, M. *et al.* High resolution Holocene record in the southeastern Bay of Biscay: Global versus regional climate signals. *Palaeogeogr Palaeoclimatol Palaeoecol* **377**, 28–44 (2013).

18. Casford, J. S. L. *et al.* A dynamic concept for eastern Mediterranean circulation and oxygenation during sapropel formation. *Palaeogeogr Palaeoclimatol Palaeoecol* **190**, 103–119 (2003).

19. Frosini, P. La Carta della precipitazione media annua in Italia per il trentennio 1921-1950. *Monografie del Servizio Idrografico Nazionale* **24** (1961).

20. Noirfalise, A. *et al*. Carte de la vegetation naturelle des Etats members des Communautés européennes et du Conseil de l’Europe. *Office des publications des Communautés Européennes* (1987).

21. Capraro, L. *et al*. Climatic patterns revealed by pollen and oxygen isotope records across the Brunhes-Matuyama Boundary in central Mediterranean (Southern Italy). Geol Soc London, Sp Publ **247** 159–182 (2005).
